# Implementation of occupational health legislation at work place, issues and concerns

**DOI:** 10.4103/0019-5278.43259

**Published:** 2008-08

**Authors:** G. K. Kulkarni

**Affiliations:** Editor IJOEM, Siemens Ltd, Kalwa Works, Thane-Belapur Road, Thane - 400 601, India. E-mail: editor@ijoem.com

In the last fifty years Indian industry has grown rapidly and more so in the last two decades. This has resulted in increased manufacturing activities, technological advancements and change in work practices. This change in business environment has profound effect on the health of working population. Thus the onus is on Occupational Health Physician to protect and promote health of working population. This can only be achieved by comprehensively implementing Occupational Health Legislations pertaining to work place.

The Occupational Health Legislation has been amended only twice in the last fifty years, one central act related to Building and other Construction workers of 1996 and The Biomedical waste (management and handling) rules of 2003 under Environment protection act of 1986 have seen the daylight, whereas in the field of Public health more than 36 different Health Legislations have come with changing time. It is only after the Bhopal disaster that legislations in the area of safety. Health and environment were forced to be reframed and notified. The concern is whether only change in legislation would result in improved Occupational health care at work place or benefits of legislation remain only on the paper is the issue. The expectation from Occupational Health Legislation would be:

**Occupational health legislation**

- Prohibit conduct injurious to workers Health
- Aims at protecting from disease and promoting positive health
- Defines resourses for occupational Health care
- Carry out medical screening, surveillance and rehabilitation
- Concerning ethical issues in occupational health care

**Current occupational health care service is because of**

- Governmental legislation for Industry, Mines, Docksand Ports and others
- Demand from the Trade Union
- Management concerns for compliance of legislation and Employee health care
- Action groups NGO like Indian Association of Occupational Health, NSC and others.

**Current factories acts – Occupational health care (Factories Act and Maharashtra factories rules as illustrative reference)**

- FA – 45- First aid appliances: Under this act and MFR- 76,77 and 78 describe First- aid facilities at work place, ambulance room. Trained First-Aiders and contents of the first-aid Box.
- MFR – 43: Drinking water certification by Health Officer for Human consumption.
- FA – 87 – MFR – 114: Medical examinations for persons employed in “25 Dangerous Operations”.
- FA – 89: Notification of Occupational diseases(29 as per Third schedule)
- MFR – 73L: Declaration of Health and safety Policy.
- MFR – 73 V: Deals with fitness for employment in form No. 6, pre-placement and Periodic Medical examinations. Maintaining Health Register in form No. 7.
- MFR – 73 W: Describes size, equipment, manpower, qualifications and facilities at Occupational health Centre
- MFR – 73X: Deals with ambulance van, emergency medicines and equipments.
- MFR – 73Z: Making available Health records to Workers.
- MFR – 116: Notice of poisoning or disease (Form no.25)

A survey was conducted amongst randomly selected 26 Industries in and around Mumbai to assess the problems faced by Occupational health Physicians in implementing legislations pertaining to Occupational health. Findings of the survey are being shared here.

## REPORTING OF NOTIFIABLE DISEASES

In the survey more than 90% of the respondent expressed that they are in conflict with themselves in reporting such cases. There is dilemma whether to report directly or through the company manager. There is also confusion and lack of clarity with respect to ESIS and Non ESIS work force. There is need for regulatory authorities to proactively guide the Occupational Health Physician which would result into effective compliance.

## INJURY AND DISEASE COMPENSATATION

Occupational Health Physicians expressed difficulties in assessing disabilities of non-amputation injuries. Hearing loss and Pneumoconiosis are areas where experts can pool in knowledge to create uniform and acceptable process protocols to deal with above issues.

## PERIODIC MEDICAL EXAMINATION

Logistic problems faced by the Occupational Health Physicians in complying with Contract Labor Medical Examination working in hazardous areas. Some times, risk communication and notifying results of medical examination to employees’ ends up with controversy, loss of trust on both sides i.e. management and trade union. Nearly 10 to 20 % of employees do not attend medical examination and hardly any action can be taken on them. The surveillance program is difficult to sustain in mining and construction sectors. The demand for change of job after risk communication increases which many times leads to friction and strained relations, and loss of trust in medical examinations. Some times business managers do not spare employees for medical examinations because of production pressures. Many felt that statutory forms need to be modified and made user friendly.

## TRAINING IN OCCUPATIONAL HEALTH AND LACK OF TECHNOLOGY SUPPORT

In the survey 69% Occupational Health Physicians felt that adequate training opportunities in Occupational Health are not provided and at work place there is lack of support in the form of Industrial Hygiene technology. Since 70% of the work currently is focused on curative mode, Occupational Health Physicians felt that they must reorient themselves to practice of Occupational Health care technology. Many felt that even the certifying surgeons were short of competency required to deliver Occupational Health Service. It is my view that there must be accreditation system in certifying the competency levels of Certifying Surgeons and IAOH can play techno-participatory role in helping the Government to bridge this gap.

## DIVERSITY OF LACK OF INTEREST BY OCCUPATIONAL HEALTH PHYSICIANS

The language and presentation of Occupational Health laws did not generate interest of reading amongst Occupational health Physicians. Many part-timers and retainers felt that lack of interest in law medicine and paucity of time are factors responsible for poor understanding of legislations. Most of the state rules are uniform but they do differ in some aspects and there is urgent need to follow common code in all states.

## JOB NOT PROTECTED BY LAW

Twelve percent of the respondents pointed out that the position of Medical Officer is not protected by law as it is for the Safety Officer. They felt that some times Occupational Health Physicians could be victimized.

## CONCLUSIONS

It is not enough that there are Occupational Health legislations but Occupational Health Physicians must put efforts to understand and will to implement Occupational Health laws pertaining to respective work places. Employer must create supportive environment for practice of Occupational Health and provide opportunities for continuous learning and training in Occupational Health. A regular dialogue must take place between Occupational Health Physicians, Safety professionals and local factory inspectorate authorities to clear doubts seek advice and bring in role clarity. Review of legislation is required once in five years and Law makers must seek feed back from Occupational Health Physicians and other sources. Governments can involve NGO's like Indian Association of Occupational Health as Member of the review committee on Occupational Health legislations. I am sure this is only a tip of the iceberg as far as issues are concerned and many more need to be addressed about which readers can write to us that can be highlighted in the future articles.

